# Suffering and Care of 0–12 Year-Old Children Exposed to Intimate Partner Violence: Making Clinical Forensic Data Talk

**DOI:** 10.3389/fpsyt.2022.805097

**Published:** 2022-04-25

**Authors:** Lyne Dessimoz Künzle, Anne Cattagni Kleiner, Nathalie Romain-Glassey

**Affiliations:** ^1^Department of Psychiatry, Lausanne University Hospital, University of Lausanne, Lausanne, Switzerland; ^2^University Center of Legal Medicine, Lausanne University Hospital, University of Lausanne, Lausanne, Switzerland; ^3^Institut et Haute École de la Santé La Source, Lausanne, Switzerland

**Keywords:** intimate partner violence, child abuse, IPV exposure, clinical forensics, domestic violence, child, care, suffering

## Abstract

Children's exposure to intimate partner violence (IPV) is a widespread phenomenon that can have detrimental consequences on their health and well-being. This study examined how clinical forensic consultation data of adult victims of IPV might provide information on the potential suffering of children exposed to IPV, the duration of exposure and the knowledge of the situation by the professionals with whom those children were in contact. Data were collected from the consultation files of 112 adult victims of IPV who consulted the Violence Medical Unit at the Lausanne University Hospital (Switzerland) in 2014, and who were parents of children aged 0 through 12. Descriptive quantitative and qualitative analyses were performed. Symptoms of suffering, such as dysregulation of instinctual functions and developmental, behavioral or emotional difficulties, were reported for nearly one-third of the victims' children. Children's exposure to IPV often started around their birth and about four in 10 children had been exposed for three years or more. Health and childhood professionals were unaware of the exposure for the vast majority of the children. Clinical forensic data can be useful in providing information on the suffering and care of children exposed to IPV. Their suffering took the form of a non-specific posttraumatic symptomatology and therefore might be difficult to detect. It is necessary to make professionals and parents aware of the fact that IPV can have a harmful impact on children's health and well-being, and to encourage health professionals to consider the possibility of IPV when facing such symptoms.

## Introduction

Children's exposure to intimate partner violence (IPV) is a form of child abuse (1–3). Exposure to IPV is not restricted to children having seen or heard violence. Indeed, living in a home with a parent victim of IPV is the criteria that defines exposure, as children can also experience the consequences of a physical aggression or live in a climate of coercive control (4–8). IPV exposure prevalence data are scarce in Switzerland, where the present study took place. However, a 2017 non-representative survey of 8,317 17 and 18 year-old students revealed that about one in five had ever observed physical violence between their parents (9). Even if not representative of all 17 and 18 year-old children in the country, these results are similar to those of two prevalence studies in the United States and the United Kingdom, both of which measured a lifetime IPV exposure rate of about 25% among teenagers and young adults (10, 11). Thus, children's exposure to IPV is a widespread phenomenon.

Living in a home with IPV is considered one of various possible adverse childhood experiences (ACEs), such as living with a parent suffering from a psychiatric pathology or an addiction, or being a victim of psychological, physical or sexual abuse, and which are known to potentially have short- and long-term detrimental consequences on health and well-being (12, 13). Moreover, experiencing multiple ACEs exponentially increases physical and mental health risks throughout life (14, 15). The negative impact of IPV on the health and development of children has been documented (16, 17) and linked to emotional and behavioral problems in children (18). In addition, exposure to IPV puts children at higher risk of experiencing other forms of abuse (19).

The first known protective factor against the consequences of IPV exposure for children is stopping the violence (20). However, research has also shown that a warm relationship to a caring adult, usually the mother, is an important protective factor (19), as well as spirituality, social support and emotional intelligence (21). In particular, self-regulation skills have been linked to adaptive functioning in children exposed to IPV (22). It is therefore crucial that exposure to IPV be detected for these children to receive proper care based on those findings.

For now, detection of children's IPV exposure in western countries is mostly achieved through the victimized mothers or other caregivers, usually when seeking help following IPV or through mental health services, while direct detection through children is more rare (23). Indirect detection routinely occurs at the Violence Medical Unit (VMU) of the Lausanne University Hospital in Switzerland, by the way of clinical forensic consultations for adult victims of violence. The data collected by the VMU from adult patients provide key information on children exposed to IPV to the Hospital's Pediatric Department Child Abuse and Neglect Team (CAN Team), which can then conduct assessments of individual situations. However, to the authors' knowledge, no studies have explored the information that could be retrieved from clinical forensic data to report on the potential suffering of children exposed to IPV as a group, and on professional awareness of those children's situation.

The first objective of the present work was to explore how clinical forensic consultations of IPV victimized parents can provide information on the suffering their children may experience and the duration of their exposure to IPV. The second aim was to use these data to assess the extent to which professionals in contact with these children are aware of those children's situations.

## Methodology

### Design

This work consisted of retrospective quantitative and qualitative analyses of clinical forensic patient files from the VMU of the Lausanne University Hospital in Lausanne, Switzerland. The results presented in this paper emerged in the context of the first author's medical thesis, which was carried out between September 2018 and September 2020 (24). The thesis itself was a component of a larger study on children's exposure to IPV (25).

### Population

The VMU offers clinical forensic consultations to adult victims of interpersonal violence, of which IPV-motivated consultations account for about a third. During the consultations, the VMU nurses or physicians systematically collect limited but key information from victimized parents on children exposed to IPV. That information is transmitted to the CAN Team, which will then make an assessment of the situation of violence, taking into account the seriousness of the acts, the potential long-term consequences on the development of the child and the ability of the family to remedy the situation (26).

All 117 files of IPV victimized adults who consulted the VMU in 2014 and who were parents (or step-parents) of children aged 0 through 12 were reviewed. After exclusion of files of pregnant women with no other children, as well as the files of parents whose children were living abroad, 112 files remained for analysis. The IPV victimized parents were mothers (or the female partner of the father in seven situations) or fathers. Included were the files of two partners who had consulted the VMU as victims for the same violent event. For ease of reading, all of the victimized female adults will hereafter be referred to as victimized “mothers,” and all victimized adults as “victimized parents.”

Only one year's data were analyzed because of the thesis's time constraints. The year 2014 was selected since this was the most recent dataset available already extracted in the context of the larger study that examined data from 2011 through 2014 (25). The rationale for focusing on 0–12 year-old children was that this was the age group most represented in the main study. Moreover, teenagers' exposure to IPV was the focus of another segment of the aforementioned larger study. The data used for the analyses thus concerned 167 children. Most children were those of the victims. However, eight children were the perpetrators' children who cohabited with their fathers' victimized partners. Children for whom both parents had consulted the VMU (two children) were only included once in the analyses.

### Data Collection

Before conducting a clinical forensic physical examination, nurses or physicians interview victims using a semi-structured questionnaire as part of the VMU consultation. For the purpose of this study, the following routinely collected data were systematically extracted from the patients' files for analysis: sex of the victim; number of children in the family; children's sex and date of birth; whether each child attended daycare or school, was followed by a pediatrician, or was seen by a psychologist, a child psychiatrist or any other professional; and whether professionals in contact with each child were aware of the situation. Information on the year the physical violence had started and on any previous history of any form of violence was also retrieved. Finally, the answer to the question “How is the child doing?” was extracted. Therefore, all data concerning the children are based on the victimized parents' accounts as reported by the clinical forensic nurses or physicians, but also as found in the communications between the VMU and the CAN Team professionals.

### Analysis

#### Quantitative Analyses

The primary quantitative data were mostly extracted from the VMU's Access database. Some data, not entered in the database, were manually entered into Excel (e.g., the birth year of each child). Other quantitative data were generated by transforming text data (e.g., to estimate the duration of IPV). The following variables were derived from the raw data:

*Child's age*—Approximate age was calculated from subtracting the birth year from the consultation year (2014). If the birth year was also 2014, age was noted as “less than 1 year”.

*Year of onset of physical violence—*This variable was created based on the information in the clinical forensic report, which is usually phrased as follows: “Mrs./Mr. indicates that physical violence began….” When the victim indicated that there was no history of physical IPV by the same perpetrator, 2014 was recorded as the year of onset.

*Duration of physical IPV*—This variable was computed by subtracting the estimated year of the onset of physical IPV from the year of the consultation (2014).

*Duration of child's physical IPV exposure*—When the physical violent event that motivated the consultation was the first one, the duration of exposure was noted as “first exposure.” It was noted as “less than one year” when the onset of physical IPV occurred in 2014 but was prior to the violent event that motivated the consultation, or when the child was born in 2014 but the violent event was not the first one. Otherwise, it was either equal to the child's age when the year of onset of physical IPV was equal to or anterior to the year of birth, or equal to the duration of physical IPV when the year of onset was posterior to the year of birth.

*Age at first exposure to physical IPV*—When the violent event that motivated the consultation was the first one, age at first exposure is equal to the child's age. Otherwise, when the duration of physical IPV was equal to or greater than the child's age, physical IPV was considered to have occurred before or around the birth of the child, and age of first exposure was therefore noted as “before or at birth.” When the duration of IPV exposure was less than the child's age, age at first exposure was calculated by subtracting the duration of physical IPV from the child's age.

The decision to limit to physical violence the analyses of duration of children's IPV exposure and of age at first exposure was motivated by the fact that it is easier for victims to identify and date a first physical aggression than the onset of other types of IPV, such as psychological violence.

*Educational and therapeutic environment of the child*—Victimized parents were also asked which professionals were in regular contact with their children, such as daycare professionals, schoolteachers, pediatricians, psychologists or child psychiatrists, other therapists and social workers. Percentages were then computed based on that information.

*Professional knowledge of IPV—*Victimized parents were systematically asked whether the professionals in the educational and/or therapeutic environment of their children were aware of the violence. Percentages were then computed based on that information.

All quantitative analyses were computed using Microsoft® Excel® 2016.

#### Qualitative Analyses

Qualitative analyses to describe any symptoms of child suffering were based on the answers to the question “How is the child doing?” recorded in the VMU's paper file, and from correspondence with the CAN Team. Some parents declared that their children were doing well and others described some difficulties, that they or health or childhood professionals had recognized. Symptoms of suffering were identified from what the parents reported and organized into categories using common psychiatric terminology. The use of these categories allows clear description of the range of difficulties experienced by these children. However, it is not an attempt to make a diagnosis, which would not be feasible based on the limited information available and without conducting an assessment of the children. This is why the term “difficulty” is used, and not “disorder.” Additionally, categories of symptoms are presented by age groups, as the mode of expression of these symptoms differ in non-school-age and school-age children and because the professionals with whom they may be in regular contact differ. Each category of symptoms identified in this population is followed by quotations in the presentation of the results. The aim is to illustrate the singularity of the expression of suffering and to provide nuance to categories that are, by nature, reductive.

## Results

### Characteristics of IPV Victimized Parents and Their Children

All but two of the victimized parents consulted the VMU following a physical aggression from their intimate partner. These two parents of four children had reported psychological violence only, with no history of physical violence.

Demographic characteristics of IPV victimized parents and their children are presented in Table 1. A large majority of the parents were mothers (84%) and a minority were fathers (16%). At the time, three women were pregnant.

**Table 1:** Characteristics of IPV victimized parents and their children.

	***N***	**%**
Parents	112	100%
Sex		
Female	94	84%
Male	18	16%
Children	167	100%
Sex		
Female	79	47%
Male	88	53%
Age group
Non-school-age	70	42%
School-age	97	58%
Siblings
Yes	111	66%
No	56	34%

Of the 167 children aged 0–12, 53% were boys and 47% were girls. Forty-two percent were non-school-age children (0–4 years-old), and 58% were school-aged (5–12 years-old). A third (34%) were only children, while the others had siblings.

### Symptoms of Child Suffering

According to the victimized parents, almost one-third of the children (31%) showed symptoms of suffering: 33% among non-school-age children and 29% among school-age children. The symptoms found in children belonging to the two age groups are listed below, in decreasing order of frequency, and are summarized in Table 2.

**Table 2:** List of symptoms^*^ in children as reported by their parents, in descending order of frequency and by age group.

**Non-school-age children** ***N* = 70**	**School-age children** ***N* = 97**
- Dysregulation of instinctual functions (sleep disturbance, appetite disturbance, change in crying) (15)	- Emotional difficulties (sadness, fear, worries, depression) (13)
- Behavioral difficulties (agitation, aggressiveness) (9)	- Behavioral difficulties (agitation, aggressiveness, nervousness, inhibition) (11)
- Developmental difficulties (language and/or psychomotricity) (3)	- Learning difficulties (hindrance to school learning, lack of attention, memory problems) (7)
- Disturbance during separations (3)	- Dysregulation of instinctual functions (sleep disturbance, appetite disturbance) (6)
- Concentration difficulties (1)	- Enuresis (3)
- Tendency toward somatic diseases (1)	- Tendency toward somatic diseases (2)

* *In response to the question: “How is the child doing?” Each child could present more than one symptom. (n) = number of children presenting the symptom*.

#### Non-school-age Children

Victimized parents of non-school-age children mentioned dysregulation of instinctual functions such as sleep, appetite, or crying. Difficulties in falling asleep, waking up at night, or having nightmares were mentioned. Appetite was decreased in one situation. Changes in daytime and nighttime crying were reported.

*A victimized father reported about his 2-year-old son: “It's hard to get him to sleep at night, he has to fall asleep with me.”*

*Another victimized father reported about his 4-year-old son: “He has nightmares from time to time, and cries at night.”*

*A victimized mother declared about her 2-year-old daughter: “Since she arrived at the shelter, she eats little, wakes up and calls me during the night.”*

In the following examples, behavioral disturbances were related. These included agitation or increased aggression.

*A victimized mother reported about her 4-year-old son: “His teachers describe him as disturbed and disruptive.”*

*A victimized mother said of her 4-year-old son: “He can hit me.”*

Developmental difficulties, such as with language, were also highlighted. In another situation, a global delay in psychomotor development was described:

*A victimized mother reported that her 1-year-old son “is slow to develop.”*

Disturbances during separations were reported:

*A victimized mother related that her 4-year-old daughter “is sometimes difficult when it comes to going to the father's house.”*

Concentration difficulties were reported:

*A victimized mother said of her 4-year-old son: “He hasn't been sleeping well since the incident, he wakes up during the night and cries. He has trouble concentrating.”*

A tendency toward somatic affections (bronchiolitis and repeated ear infections in particular) was described as well.

#### School-Age Children

In school-age children, victimized parents reported emotional difficulties such as the expression of sadness, fear, worries or depression, including one situation with verbalized suicidal ideations:

*A victimized mother said about her 6-year-old daughter: “Her teacher describes her as sad and not doing well.”*

*Another victimized mother said of her 12-year-old son: “He is afraid of his father. He is traumatized by the armed police intervention.”*

*A victimized mother reported that her 6-year-old daughter “is worried, nervous. She is afraid of her father.”*

*A victimized father declared being very worried about his 12-year-old son because he had expressed suicidal thoughts in the past*.

Other victimized parents noted behavioral difficulties such as nervousness, inhibition, agitation, and violence toward peers:

*One victimized mother reported that her 6-year-old daughter's schoolteacher thought she was stressed out*.

*Another victimized mother said of her 10-year-old son: “He is a very quiet child. He is reserved.”*

*A victimized stepmother of a 7-year-old boy and a 9-year-old girl said that she often finds them “very excitable, acting out, not getting along with each other and hitting each other.”*

*A victimized mother reported about her 6-year-old daughter: “She has become more aggressive since the last events.”*

Learning difficulties in the form of hindrance to school learning, lack of attention or memory were related:

*A victimized father said about his 7-year-old son: “He has difficulties in school that have gotten worse since the separation.”*

*Another victimized mother reported about her 12-year-old son: “At school he forgets things, he is in his thoughts.”*

Dysregulation of instinctual functions such as sleep (disturbed sleep) or appetite (disturbance, overweight, obesity) were related:

*A victimized mother said of her 7-year-old daughter: “Since the last episode, she doesn't sleep as well as she used to, she has trouble falling asleep, she wakes up during the night and seeks refuge in our bed.”*

Enuresis and a tendency toward somatic affections (eczema) were also noted.

### Duration of Physical IPV Exposure

#### Duration of Physical IPV

Nearly 9 out of 10 victimized parents (88%) reported a history of physical IPV in the relationship by the same perpetrator in the past, and about 1/10th (12%) reported that this was the first. This did not preclude other forms of violence (psychological, economic, or sexual) in the past.

Victimized parents with a history of physical violence frequently reported other forms of violence by the same perpetrator in the past. Psychological violence, such as threats, insults, or denigration, was declared in nearly one out of two situations (51%). Economic violence was specifically reported in a little more than 1/10th of the situations (11%), and sexual violence in 6% of the situations (all victims were women).

When the physical aggression that motivated the consultation was the first one (12%), previous psychological violence was reported as well in about half of the situations, sexual coercion in one situation, and psychological violence was accompanied by economic violence in another one.

#### Duration of Children's Physical IPV Exposure

**Figure 1:**
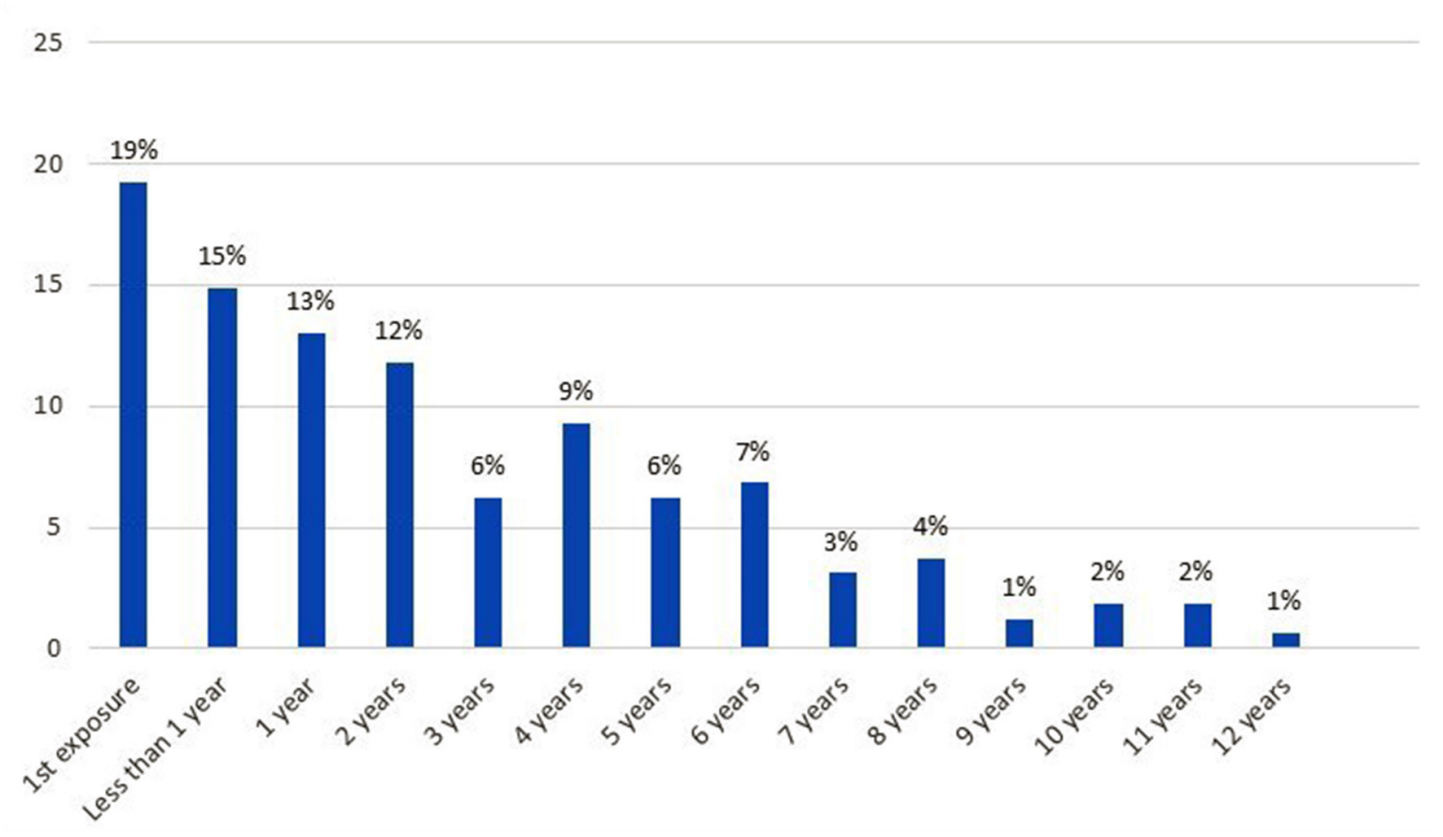
Duration of children's physical IPV exposure (*N* = 161. The information was missing for two children).

Figure 1 shows the duration of exposure of children to physical IPV, which can go up to 12 years. While the aggression that motivated the consultation corresponded to the first exposure for 19% of the children, 40% had been exposed for less than one year to two years, and 41% for three years or more (calculated from Figure 1).

#### Age of Children at First Physical IPV Exposure

**Figure 2:**
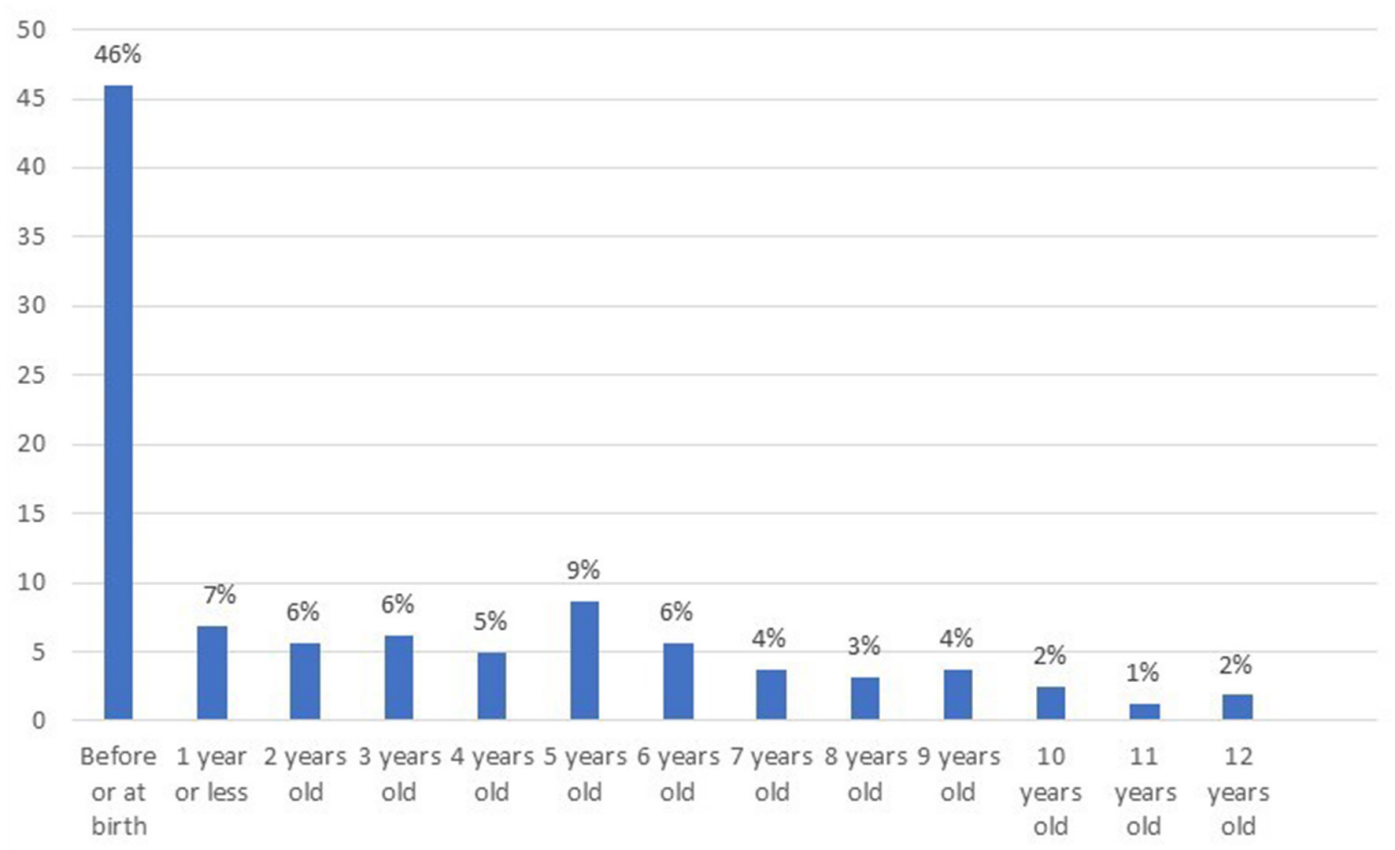
Children's age at first physical IPV exposure (*N* = 161. The information was missing for two children).

Figure 2 details the estimates of the ages at which children were first exposed to physical IPV. Physical IPV started before or around birth for a majority of the children (46%), who therefore had been exposed their entire lives.

### Visibility of IPV Among Health and Childhood Professionals

The types of health and childhood professionals in contact with the children and aware of IPV are presented in Table 3. Almost half of non-school-age children (47%) attended daycare. Early childhood educators of nearly one-third of those children (30%) were aware of the violence. All school-age children attended school. Schoolteachers of less than one in 10 children (8%) knew about the violence.

**Table 3:** Awareness of IPV situations by professionals in contact with children.

**Type of professionals**	**Children in**		**IPV situations**	
	**contact with**		**known by these**	
	**professionals**		**professionals**	
	***N***	**%**	***N***	**%**
- Early childhood educators^*^	33	47%	10	30%
- Teachers^**^	97	100%	8	8%
- Psychologists, child psychiatrists	26	16%	7	27%
- Pediatricians	161	96%	26	16%

*N = 167*.

* *Data relative to non-school-age children only (N = 70)*.

** *Data relative to school-age children only (N = 97)*.

Sixteen percent of all children had received or were receiving psychological or psychiatric care. Psychologists or child psychiatrists were aware of IPV for a little over a quarter of the children they followed (27%).

According to the victimized parents, 96% of children were followed by a pediatrician. Pediatricians were aware of the violence for 16% of them.

Additionally, two children were receiving care from a speech therapist, another one from an early childhood nurse and another one received services from an itinerant educator. Two out of the other four aforementioned professionals were aware of the violence and that information was missing in one situation (not shown in table).

## Discussion

The results of this study showed that clinical forensic data can provide useful information on the suffering of children exposed to IPV. About one-third of the children included in the study presented symptoms of suffering. The results also revealed that the duration of exposure could be counted in years and that children were often exposed since or before birth. In parallel, few professionals in contact with those children were aware of the situations they were experiencing at home.

### A Probable Link Between IPV Exposure and Symptoms of Suffering

The symptoms described in this study are not based on any formal assessment but on a single and general question asked to victimized parents about their children, and so do not constitute a diagnosis. Thus, strictly speaking, comparing their global proportion to prevalence rates from other studies would not be appropriate. However, it is noteworthy that the proportion of emotional and behavioral difficulties in our school-age children population (21%, calculated from row data, not shown in tables) is closer to the one found in 10-year old children exposed to IPV (19%) than the one of same-age children not exposed to IPV (10%), as measured by the Strength and Difficulty Questionnaire, answered by mothers, in a recent study by Gartland et al. (27).

The symptoms of child suffering reported by the victimized parents in our study correspond to what is generally described in the literature, namely clinical and psychological repercussions of children's exposure to violence in the form of a non-specific posttraumatic symptomatology with variations according to the child's age. Indeed, Mueller and Tronick's review examining the evidence of the impact of IPV exposure from the perinatal phase through early childhood found clear physical and psychological adverse influences (28). They report the identification of symptoms similar to the ones found in our study, which they relate to posttraumatic symptomatology, such as difficulties in eating and sleeping, mood problems, or increases in irritability and crying.

Other studies have found a significant influence of IPV exposure on the neurobiological development of children, with psychosomatic and psychotraumatic suffering, with children 5 years-old and younger presenting regressive behaviors such as clinging to a reference person, and increased aggression and nightmares (29). These symptoms are similar to the ones found in our study, namely *disturbances during separation, sleep disturbances* and *aggressiveness*. Neurological and language delay in infants and toddlers have also been highlighted (30) and could correspond to the *developmental difficulties* found in our population of non-school-age children. The main symptoms identified in the present study were also highlighted in a longitudinal study comparing children at age 10 exposed and not exposed to IPV (27). Indeed, Gartland et al. found that children of mothers who had reported IPV were twice as likely to experience emotional and behavioral difficulties, impaired language skills and sleep problems. Our results showed that non-school-age and school-age children expressed their suffering differently. Indeed, non-school-age children were more inclined to modify the amount of their crying or to cling to their reference figures, while other emotional and learning difficulties appear in school-age children. This partly corresponds to findings of a study examining posttraumatic stress disorder (PTSD) symptoms among 1 to 7 years-old children exposed to IPV: while in non-school-age children symptoms were more related to affective dysregulation, school-age children begin to show more cognitive dysregulation and behavioral difficulties in response to being exposed to IPV (31).

Although the aim of the present study was not to make links with the diagnosis of PTSD, in light of the literature described above, it is noteworthy that the symptoms of suffering found in our population of non-school-age children correspond to several criteria as described for PTSD diagnosis in the DC:0-5 (32) under the group “onset or intensification of signs of increased arousal.” Namely, “sleep problems” can be related to the *dysregulation of instinctual functions* found in our study, and “increased irritability, outbursts of anger or extreme fussiness, or temper tantrums” to the *behavioral difficulties (agitation, aggressiveness)*. Additionally, “difficulty concentrating” described in the manual was also found in our study. The DC: 0-5 also points out that other associated features can support the diagnosis of PTSD, such as the appearance of “separation anxiety” or a “developmental regression or loss of previously acquired skills.” These features can also be found in our population of non-school-age children under the symptom categories *disturbances during separations* and *developmental difficulties*. With regard to the symptoms of suffering found in our population of school-aged children, several can be linked to criteria for PTSD as well, according to the DSM-5 classification (33). *Emotional difficulties* can fall under the criterion “negative alterations in cognition and mood,” *behavioral difficulties* and *dysregulation of instinctual functions* under the criterion “marked alterations in arousal and reactivity.”

Our results, similar to those of Graham-Bermann and Perkins (34), revealed that the majority of the children were exposed to physical IPV before birth or in the first few months of their lives. Studies have shown that children who have been exposed to IPV at a very young age seem particularly vulnerable and present a higher risk of emotional suffering and behavioral difficulties than the others (35, 36). Moreover, most of the children in the present study had been exposed to IPV for several years, and it is known that the greater amount of violence to which children are exposed tends to increase the probability of symptoms in children (34). It is therefore likely that a longer duration of IPV exposure will increase the likelihood of adverse effects in children.

However, not all parents reported symptoms of suffering in their children. As noted in the introduction, individual resilience factors to IPV exposure have been identified in the literature, such as self-confidence, self-regulation, and pro-social skills (19, 31). Other reviews show protective factors at the family and environmental level, for instance a relationship of trust with a reference figure or community support (19, 37, 38). It seems that the influence of these factors modulates the individual response to exposure and explains at least in part the variability in the expression of suffering among children.

Our results seem therefore to be in line with what is described in the literature. However, the picture is broad, touches on different registers of mental illness, and therefore, is not very specific. The difficulty in conceptualizing a possible syndrome that would help in detecting exposure to IPV in children seems to be due to various factors. For very young children, it is mainly the difficulty of communicating their suffering to those around them because of the lack of verbalization and language linked to their stage of development. Older children may have an avoidance tendency and feel embarrassed about the family situation (39). Protective factors can also play a moderating role in the child's individual response to IPV, as discussed above. Moreover, several studies point to the influence of co-occurring forms of socioeconomic vulnerabilities (25, 40) or maltreatment (16, 17), such as direct physical abuse, which can also complicate the analysis of the direct impact of IPV.

### A Phenomenon With Little Visibility Among Health and Childhood Professionals

Our results also show that children's IPV exposure was rarely known by professionals in regular contact with them^1^. Early childhood educators were more often aware of the situation than school teachers. This could be explained, at least in part, by the high frequency of contacts and exchanges between parents and early childhood educators, whereas school-age children can go to school without needing to be accompanied by their parents, thus limiting teacher-parent contacts. Münger and Markström (41) examined whether and how early childhood educators and schoolteachers identified children exposed to IPV. Their work revealed different elements that might prevent identification and/or reporting, such as insufficient knowledge of the phenomenon or how to identify its signs, the recognition of it as child abuse, or the influence of their vision of school duties in relation to family violence.

Psychologists or child psychiatrists were aware of IPV exposure for a little more than a quarter of the children they followed. This proportion is relatively low given the specialization of these professionals. This could be due in part to the fact that the symptoms of children exposed to IPV are not very specific. According to victimized parents, pediatricians were rarely aware of the situation. It is likely that their lack of awareness is at least in part due to the reluctance of professionals to discuss the subject, as suggested by the findings from a study in which private practice pediatricians were interviewed on the topic of detection of child abuse (42). These professionals confided their fear that overly intrusive questioning could lead parents to change doctors, with the risk of losing contact with the children. A lack of training on IPV among health professionals in general can also probably explain the limited knowledge of pediatricians and mental health professionals about these individual situations. Indeed, at the national level, a recent governmental report points out the need to better integrate the topic of domestic violence in the training of health care professionals (43). Moreover, it is also likely that some parents are not aware of the impact IPV exposure can have on their children, or that IPV is not mentioned by parents because it remains a taboo subject for victims (44). Finally, some authors have suggested that parents do not always have an accurate appreciation of their children's suffering, and that their children may also try to mask their symptoms in order not to worry and protect their parents (39, 45).

### Implications for Practice

Professionals should be made aware of the traumatizing impact that IPV exposure can have on children, all the more so given the high prevalence of this phenomenon. Because the expression of children's suffering in relation to IPV exposure is non-specific, it is important for health and childhood professionals to have a sound knowledge of posttraumatic symptomology and its nuances according to children's age. Then, facing any signs or symptoms of suffering, it is necessary that they consider the possibility of exposure to IPV (46). Finally, clinicians should have in mind that children exposed to IPV are at higher risk of exposure to other forms of abuse (19, 46).

The lack of awareness of IPV among health and childhood professionals found in the present study also shows the importance of the detection of children's IPV exposure in adult clinical consultations, such as occurs at the VMU. The efficiency of such a detection is however contingent on a good and institutionalized coordination with a pediatric service. This collaboration helps determine which information to collect during the adult consultation, and will ensure that adequate care and support are provided to children following detection (26).

### Study Limitations

The main limitation of this study is that children have not been directly assessed, and that this kind of data might be biased in that it reflects the perspectives of the victimized parents. The latter may feel a strong sense of shame or may not be aware of their children's suffering, and it is possible that not all of the children's suffering was conveyed during the consultations. However, these data present the advantage of providing useful information in the absence of data from direct detection. Another limitation is that the data analyzed pertain to VMU patients only, who cannot be considered as representative of all IPV victimized parents. By extension, the children included in the analyses do not represent all 0–12 year-old children exposed to IPV. Finally, even if the data are 8-years old, there is nothing to suggest that results would be any different today, especially in relation to duration of exposure and symptoms. Also, there has been neither awareness-raising campaigns on exposure to IPV nor the publication of professional guidelines in Switzerland in the past few years that would make professionals' awareness of IPV situations any greater today.

### Conclusion

The analyses conducted provide numerous clues confirming the evidence from international studies that children's exposure to IPV may have an impact on the development of these children. The majority of the children were exposed since or before birth, making them particularly vulnerable and more at risk for emotional distress and behavioral difficulties than others. These children are paradoxically not very visible. It appears that the suffering of children may be difficult for professionals to grasp, as its symptoms are not very specific. It is then all the more difficult to assess their needs and provide them with support. The information obtained in the context of clinical forensic adult consultations can make an important contribution to the understanding of children's exposure to IPV. If it seems necessary to pursue efforts to prevent IPV, a major awareness-raising effort concerning the impact of this violence on children is crucial for professionals working with children and their parents. Further research, focused on the needs of these children, from their own viewpoints, could support it. Increased awareness would allow for a better appreciation of the situation of and therefore a better care for children exposed to IPV.

## Data Availability Statement

The original contributions presented in the study are included in the article/supplementary material, further inquiries can be directed to the corresponding author.

## Ethics Statement

In accordance with the research protocol (2017-01736) approved by the Swiss Ethics Committee (CER-VD), the retrospective use of data concerning patients was authorized without the explicit consent of the patients, on the condition that all personal data be anonymized.

## Author Contributions

NR-G is the head of the VMU and responsible for data collection. LDK and NR-G designed this work. LDK and ACK conducted the literature review. LDK underwent the analysis and drafted the manuscript. All authors critically reviewed the structure, the content of the manuscript and approved its final version for publication.

## Funding

Open access funding provided by University of Lausanne.

## Conflict of Interest

The authors declare that the research was conducted in the absence of any commercial or financial relationships that could be construed as a potential conflict of interest.

## Publisher's Note

All claims expressed in this article are solely those of the authors and do not necessarily represent those of their affiliated organizations, or those of the publisher, the editors and the reviewers. Any product that may be evaluated in this article, or claim that may be made by its manufacturer, is not guaranteed or endorsed by the publisher.

## Footnotes

^1^The lack of studies with similar measures prevents comparing our findings with others.
